# 3B circumscribed masses: to assess or not to assess?

**DOI:** 10.1038/sj.bjc.6604500

**Published:** 2008-08-05

**Authors:** F Bonetti, E Manfrin

**Affiliations:** 1 Department of Pathology, Verona University, Verona, Italy

Sir,

We read with great interest the paper by Farshid *et al* (2008) recently published in the *Br J Cancer.*

The authors address a complex problem that is the daily experience of professionals involved in breast cancer screening programmes worldwide.

In fact, although radiologically suspicious lesions have well-recognised guidelines intended to determine the nature of the lesion, there is no definitive agreement as to how a discrete mass of indeterminate/equivocal significance (3B) should be approached.

In particular it is not very clear whether these benign-looking lesions should be followed up with mammography or whether they warrant a more ‘aggressive’ approach, using fine needle aspiration cytology or core biopsy, to define more precisely their nature.

Using a stepwise approach to the use of FNAB and core biopsy in 1183 screen-detected category 3B lesions, Farshid *et al* (2008) have shown that 8.3% of these were, in fact, malignant.

We would like to add our experience with 3B-circumscribed masses observed in the Verona Breast Cancer Screening Program.

In Verona, the breast cancer screening programme is designed in a way similar to that reported by Farshid *et al* (2008), as it is based on an integrated radio-pathologic programme with on-site pathologists who perform real-time immediate assessments of FNAC samples and discuss these results with the radiologists. A stepwise approach is similarly employed with FNAC as the first-line diagnostic modality and core biopsy as the second-line modality.

In the screening at Verona, the global FNAC accuracy in defining breast lesions (PPV of 99.3%, NPV of 98.6%) (Manfrin *et al*, 2008) reflects Farshid *et al* (2008) data, confirming FNAC as a reliable first diagnostic tool for the assessment of breast lesions.

From 1999 to 2004, we have observed and cyto-histologically characterised 388 circumscribed 3B lesions, and the pathological analysis (387 FNAC and 23 core biopsies) has shown that 19 (4.89%) were, in fact, carcinomas (nine invasive ductal carcinomas of no special type, five mucinous carcinomas, one medullary carcinoma, one invasive papillary carcinoma, one invasive lobular carcinoma and two *in situ* carcinomas, one ductal type and one lobular type).

No highly aggressive tumours were observed in our series.

Thus, our findings confirm the experience reported by Farshid *et al* (2008) that cyto-histological assessment of circumscribed 3B lesions increases the number of cancers detected.

But is this useful?

In fact, there is no definite proof that the detection of more tumours, obtained with the assessment of circumscribed 3B lesions, will ameliorate the final results of the screening, namely the reduction of mortality.

Although a longer follow-up is needed to clearly define the impact on mortality, we might consider that the tumours observed in Farshid *et al* (2008), and in our experience alike, are mostly of low-grade aggressiveness. Thus, a significant impact on the reduction of mortality seems unlikely.

On the other hand, if all 3B lesions are assessed, an increase of the rate of recalls for assessment is inevitably derived.

Not surprisingly, in the general report of activity of the Verona Breast Cancer Screening (Mariotto *et al*, 2007), we observed a high rate of assessments for breast lesions, 10.9% in the first round and 5.4% in subsequent rounds, not acceptable according to the European Guidelines, and a cancer detection rate of 9.7‰ in first round and 5.1‰ in subsequent rounds, which is within the desirable standards according to the European Guidelines (Commission of the European Communities, 2006).

Thus, if the reduction of mortality is the sole and only goal of the screening, the assessment of 3B lesions should be discouraged.

On the other hand, if we consider other factors, the balance between pros and cons becomes more difficult. As so well posed by Farshid *et al* (2008), ‘many clinicians are uncomfortable with the watchful waiting approach’, as ‘delay in the diagnosis of malignancy is one of the chief factors in medico-legal disputes concerning breast disease’.

We would like to add another aspect we have observed in our daily practice, namely a sort of ‘pressure’ from the women attending the screening programme to obtain the same diagnostic quality of a breast clinic.

Obviously, many other factors should also be considered, including the cost/benefit ratio, psychological impact, and so on.

The paper of Farshid *et al* (2008) addresses this problem in a clear and documented manner and is likely to spark a fruitful debate about the best way to approach 3B lesions.
